# Use of the Local False Discovery Rate for Identification of Metabolic Biomarkers in Rat Urine Following Genkwa Flos-Induced Hepatotoxicity

**DOI:** 10.1371/journal.pone.0067451

**Published:** 2013-07-02

**Authors:** Zuojing Li, Qing Li, Lulu Geng, Xiaohui Chen, Kaishun Bi

**Affiliations:** 1 School of Medical Devices, Shenyang Pharmaceutical University, Shenyang, China; 2 School of Pharmacy, Shenyang Pharmaceutical University, Shenyang, China; Mayo Clinic, United States of America

## Abstract

Metabolomics is concerned with characterizing the large number of metabolites present in a biological system using nuclear magnetic resonance (NMR) and HPLC/MS (high-performance liquid chromatography with mass spectrometry). Multivariate analysis is one of the most important tools for metabolic biomarker identification in metabolomic studies. However, analyzing the large-scale data sets acquired during metabolic fingerprinting is a major challenge. As a posterior probability that the features of interest are not affected, the local false discovery rate (LFDR) is a good interpretable measure. However, it is rarely used to when interrogating metabolic data to identify biomarkers. In this study, we employed the LFDR method to analyze HPLC/MS data acquired from a metabolomic study of metabolic changes in rat urine during hepatotoxicity induced by Genkwa flos (GF) treatment. The LFDR approach was successfully used to identify important rat urine metabolites altered by GF-stimulated hepatotoxicity. Compared with principle component analysis (PCA), LFDR is an interpretable measure and discovers more important metabolites in an HPLC/MS-based metabolomic study.

## Introduction

As a newly emerging field of the ‘omics’ domain based on the exhaustive profiling of metabolites, metabolomics has been widely employed to monitor global metabolic changes taking place in biological systems. More recently, HPLC/MS methodology, either alone or in combination with NMR analysis, has been used to characterize large numbers of metabolites, yielding a ‘metabolic fingerprint’ of the biological system under investigation [1]–[8]. When HPLC/MS technology is used for metabolic fingerprinting [9], [10], the unique mass-charge (m/z) value and retention time of compounds are used to construct a metabolic fingerprint that will undergo statistical analysis. This procedure includes biomarker identification by multivariate analysis of metabolic data sets [11]. As with all the ‘omics’ technologies, multidimensionality is a characteristic of metabolic data [12]. Thus, the major challenges confronting researchers are the analysis of large-scale data sets produced from metabolic fingerprinting and the selection of appropriate multivariate methods to find biomarkers effectively and precisely.

As a pattern recognition method, principle component analysis (PCA) is often used in the process of biomarker detection [13]. PCA is a dimension reduction technique [14], [15]. It is of particular utility if the original dataset is multidimensional, as PCA reduces the number of features to a manageable size. The reduced dataset can then be further analyzed by cluster analysis or various classification methods [16].

However, PCA is a relatively simple and crude method when used in biomarker detection studies [17]. PCA cannot provide quantitative evidence to determine whether a particular metabolite is a biomarker, whereas mathematical-statistical methods can provide such evidence. Considering the metabolic biomarker identification problem from the perspective of metabolic fingerprinting using HPLC/MS technology, we usually study m/z values at different retention times simultaneously. Hence, the metabolic biomarker identification challenge is a multiple hypothesis testing problem. The local false discovery rate (LFDR) represents the posterior probability that the null hypothesis is true [18]. In other words, with regards to metabolic biomarker identification, LFDR is the posterior probability that the features of interest are not changed between the control and case groups at different retention times.

The LFDR is rarely employed to find biomarkers in metabolomic studies. In this study, the LFDR method was successfully applied for HPLC/MS data analysis, as biomarkers of Genkwa flos (GF)-induced hepatotoxicity were identified in rat urine. Compared with PCA, LFDR is interpretable measure and finds more important metabolites. Using the LFDR estimation method to address the problem of biomarker identification, we could not only find biomarkers but also effectively interpret them. For example, if a metabolite with an LFDR estimate of less than 0.05 is detected as a biomarker of a particular treatment, then there is a greater than 95% probability that the metabolite is truly affected by the medical treatment. However, a biomarker detected via PCA cannot provide such interpretable evidence. A novel point made in this study that one can take the LFDR estimation method into account when addressing the metabolic biomarker identification problem. From a statistical point of view, the challenge of biomarker detection is a multiple hypothesis testing problem.

## Materials and Methods

### Ethic Statement

The study was approved by the Education and Research Committee and the Ethics Committee of Shenyang Pharmaceutical University (approval # SPU20104432). Animals were maintained and experiments were conducted in accordance with the Institutional Animal Care and Use Committee, Shenyang Pharmaceutical University, and with the 1996 Guide for the Care and Use of Laboratory Animals (Institute of Laboratory Animal Resources on Life Sciences, National Research Council, National Academy of Sciences, Washington DC).

### Data Description

**Figure 1:**
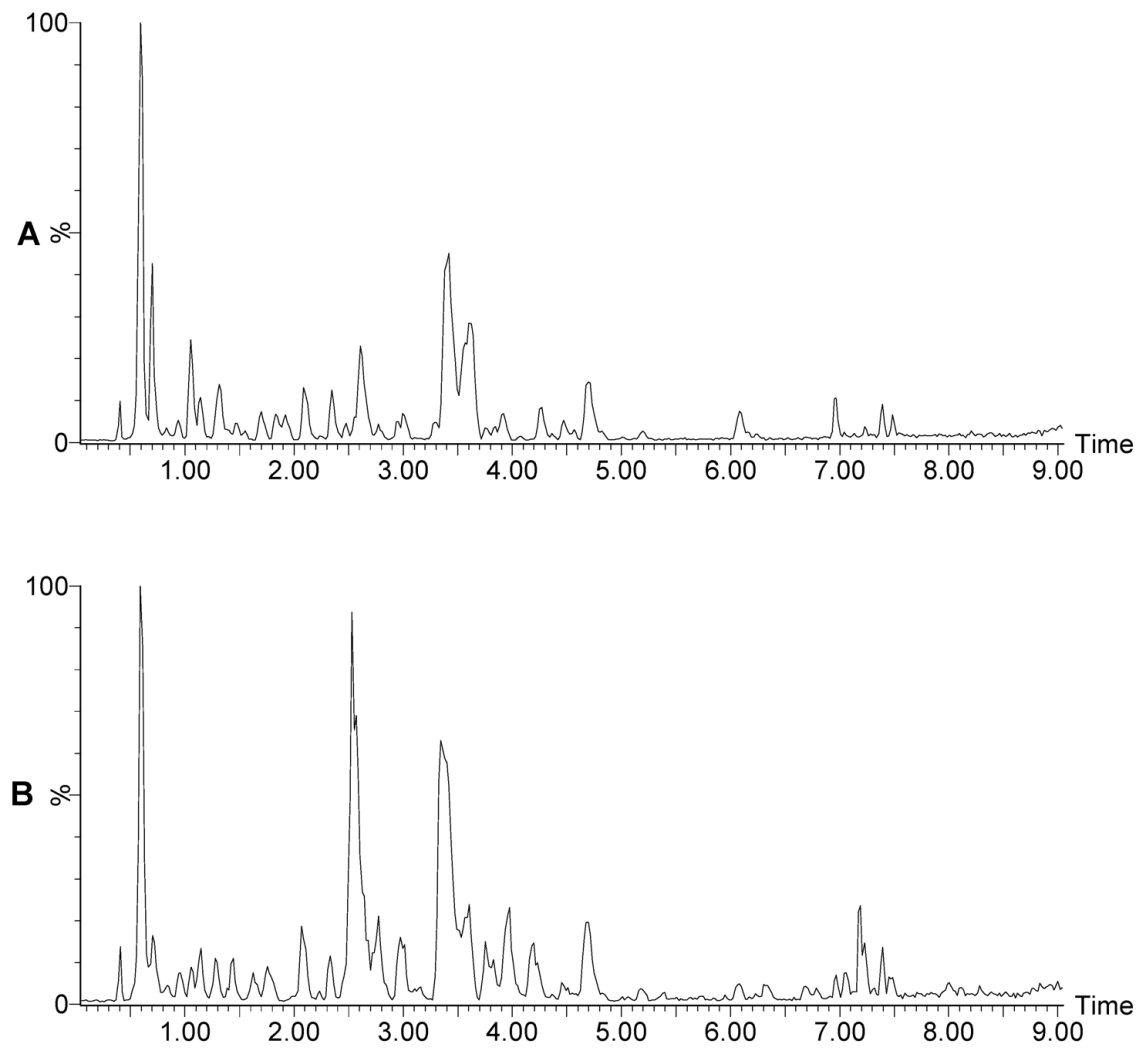
Representative positive base peak intensity (BPI) chromatograms of urine samples at 336 h post-treatment: (A) GF-treated group (B) Healthy control group [19].

Data from HPLC/MS experiments was used to study the changes in rat urine following GF-induced hepatotoxicity [19]. There were two separate data groups: GF-treated rats (case) and healthy control rats (control). Fig. 1 depicts the representative positive base peak intensity (BPI) chromatograms of urine samples at 336 hour post-treatment for the case and control groups. The main purpose of studying the effects of GF treatment was to establish a methodology for biomarker identification. The metabolomic data was imported into Micromass Markerlynx software for data preprocessing, including peak alignment and identification. The data presents the m/z values at different retention times in the case and control groups. The explanatory variable consists of metabolite-profiling data (m/z) from chromatography experiments. The response variable is ranked by retention time and m/z. Each data set contained 878 variables in which retention time changed every 0.001 min from 0.3 to 7.6 min. Each metabolite (feature) was determined by the same row data, while the differences between the control and case groups were determined by the quantitative data.

### Data Analysis Methods

#### PCA method

PCA is the most commonly used method in metabolomic data analysis [20]. Let U be an n×m matrix of m/z data denoted by (U_1_, U_2_, …,U_m_), with each being described by m descriptors. From the original set of variables U_t_, PCA constructs a new set of uncorrelated and orthogonal variables V_i_, which are linear combinations of the mean-centered variables from the original set of variables, also called loadings or principal components, that explain most of the variability of the data. For biomarker identification, the first two components are used to discriminate best between the two groups [20]. For each loading vector V_i_, the corresponding eigenvalue tells us how much of the variability of the data is explained by V_i_. We projected the data points onto the subspace spanned by the loading vectors and computed their coordinates with respect to V_i_, which is called scores. Score plot could visualize the classification of data. After the points were divided into two groups based on the scores plot, we chose the points in loading plot that were far away from the origin as potential biomarkers.

#### LFDR methods

Consider the hypothetical comparison H_0_ versus H_1_. Let π_0_ be the proportion of null hypotheses H_0_ that is true, f_0_(t) be the density function of the statistic t when the null hypothesis is true given data D, and f_1_(t) be the density function of the statistic t when the alternative hypothesis is true given data D. Therefore, the mixture density function of the data D is
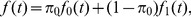
(1)


### Based on Bayesian Theory, the LFDR is Computed with the Equation



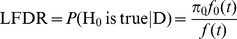
(2)where f(t) is defined in equation (1).

We presented the LFDR computational method based on Bayesian theory. However, we cannot obtain the real LFDR from equation (2) since we cannot obtain the population (i.e. the whole biomarkers). Hence the LFDR estimation method is required to address the multiple hypothesis testing problem. Efron [18], [21] introduced a semi-parametric LFDR estimation method that approximates the LFDR of the i-th hypothesis comparison, denoted as LFDR_i_, using equation
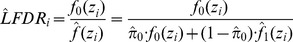
(3)where z_i_ is the z-value of the i-th hypothesis comparison obtained from p-value by normalized transformation, i.e. 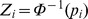, Φ is cumulative distribution function (CDF) of the standard normal distribution N(0,1) and 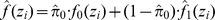 is an estimate of the mixture density function 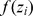 using Poisson regression. Finally, 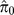 is an estimate of π_0_, which defined in (1). Efron’s method [18], [21], [22] is broadly used in gene expression data analysis because it requires a large number of hypothesis comparisons to obtain a reliable LFDR estimator. Based on microarray techniques, we can take hundreds or even thousands of genes into consideration simultaneously. However, in the GF studies, there are no such large numbers of candidate metabolites to use for biomarker identification. Therefore, it is difficult to obtain a reliable LFDR estimate using Efron’s method. A new estimation method is required to address the biomarker problem.

Let 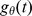 be the probability density function admitted by the noncentral distribution with noncentrality parameter value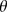. t is a statistic obtained from the data. For the hypothesis comparison i (i = 1, …, M), we defined the three-component parametric mixture model (three-component PMM) as
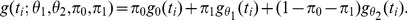
(4)


We assume that all statistics satisfy the same three-component PMM. Then, for M hypothesis comparisons
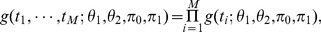
(5)where 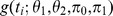 is defined in equation (4). Therefore, the log-likelihood function with the k-component PMM is



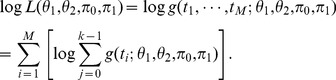
(6)The LFDR of the i-th hypothesis comparison is estimated by
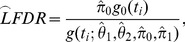
(7)where 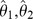 and 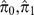 are maximum likelihood estimates of 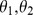 and 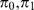 in equation (6).

The calculations performed with data analysis by LFDR estimation method are shown below.

The hypothesis comparison of the ith metabolite is 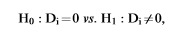


where D_i_ is the difference of m/z at retention time i. In what follows, the process of data analysis is shown.

- Compute the statistic of m/z at all retention times

Let 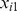,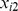, …,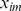 be the m/z in the control at retention time and 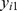, 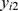, …, 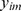 be the m/z in the case at retention time i. The statistic t_i_ at retention time i is computed by
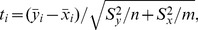
(8)where 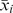 and 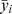 are the sample mean of m/z in the control and case, respectively, at retention time i; 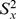
and 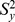 are the sample variance of m/z in the control and case, respectively, at retention time i. Therefore the statistic 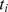 satisfies the Student’s t-distribution.

- Define the mixture density function 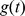

We assume all statistic t is satisfy the same three-component PPM shown in equation (4), where 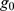 is the density function of central t-distribution; 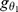
and 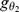
are the density function of noncentral t-distribution with noncentral parameters 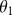 and 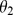.

- Estimate LFDR

The LFDR of retention time i, termed LFDR_i_, is estimated by 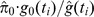, where


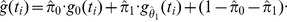
 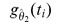;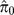,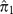,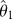,
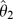


are the maximum likelihood estimator (MLE) of 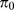,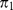,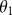,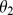 with the likelihood function 
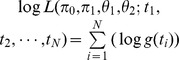
, N is the number of retention times.

#### Simulation

**Figure 2:**
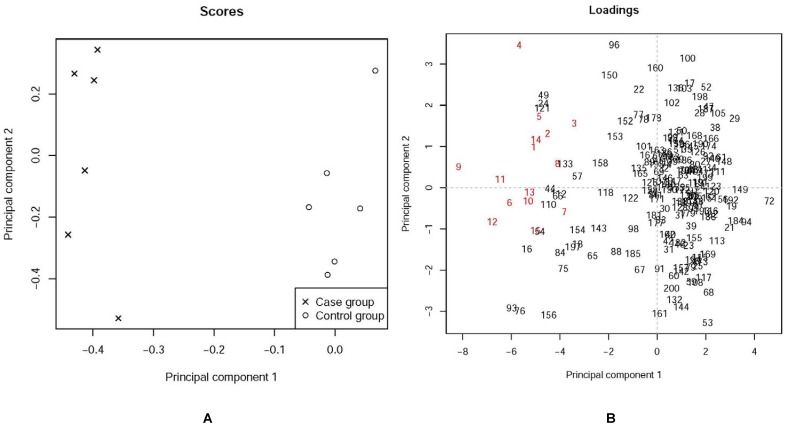
The PCA score plot and corresponding loading plot for the simulation data: (A) The score plot showing the separation between the Case group(X) and the Control group (o) (B) Loading plot for potential biomarker recognition. Metabolites 4, 6, 9, 11, and 12 are identified as potential biomarkers. Integers in red indicate biomarkers and integers in black indicate other markers.

**Figure 3:**
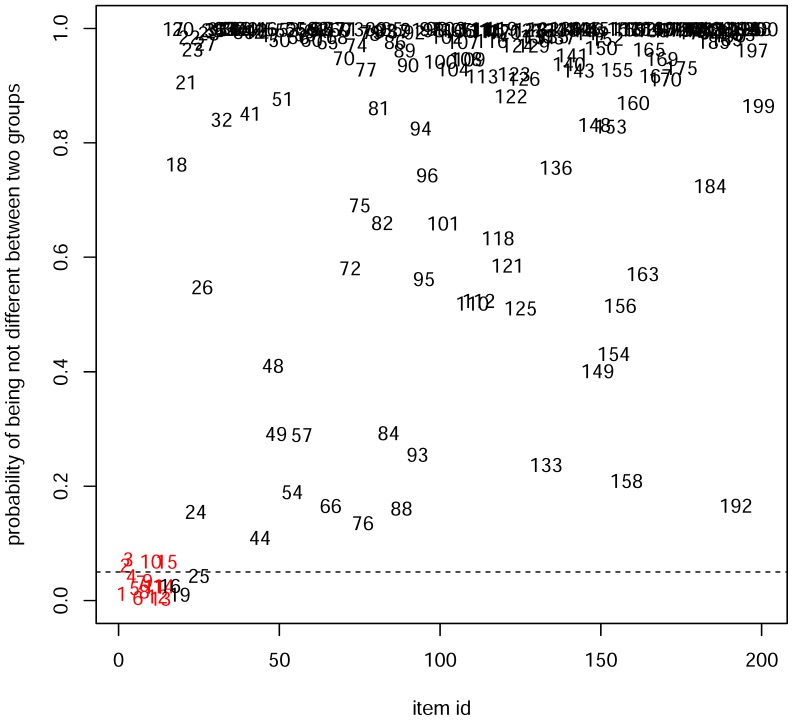
LFDR plot for simulation data of the Case group and the Control group. Integers in red indicate potential biomarkers and integers in black indicate other markers. 0.05 (dash line) is treated as the threshold for potential biomarkers identification. The integers below the dash line are identified as potential biomarkers.

To compare the biomarker identification capabilities of LFDR and PCA, a database was built. We constructed peak data for 200 candidate markers, with 15 markers identified as potential biomarkers. For each marker, 6 samples in the case group and 6 samples in the control group were used. The data is simulated as follows. For each marker of the first 15 markers which were designed as potential biomarkers, we generated 6 samples in the control group from central t-distribution and 6 samples in the case group from the non-central t-distribution with the noncentrality parameter 2. For each of the remaining 185 candidate markers, we generated 12 samples (6 for each group) from the central t-distribution. The PCA and LFDR estimation methods were used to identify potential biomarkers. The PCA score loading plots are shown in Figure 2. The LFDR plot is depicted in Figure 3. The LFDR is the posterior probability that the features of interest are not changed between the case and control groups at different retention times.

Fig. 2 shows the score and loading plots. It can be seen from the PCA score plot (Fig. 2A) that a clear separation of the control and case groups was achieved. In Fig. 2B, a marker is considered as a potential biomarker if its corresponding point in the loading plot is far enough away from the origin. Thus, metabolites 4, 6, 9, 11, and 12 are potential biomarkers, while metabolites 1–3, 5, 10, 13, 14, and 15 are not potential biomarkers. The LFDR of markers 1, 4–9, and 11–14 is less than 0.05 (Fig. 3), which means the probability that markers 1, 4–9, and 11–14 are not changed is less than 0.05. Thus the metabolites 1, 4–9, and 11–14 are potential biomarkers using the LFDR method. It can be concluded that the LFDR method is better at identifying biomarkers than the PCA method.

## Results

The PCA analysis was carried out by an R package. An R package named MLE-LFDR [23] was developed by the authors to estimate LFDR to identify potential biomarkers.

**Figure 4:**
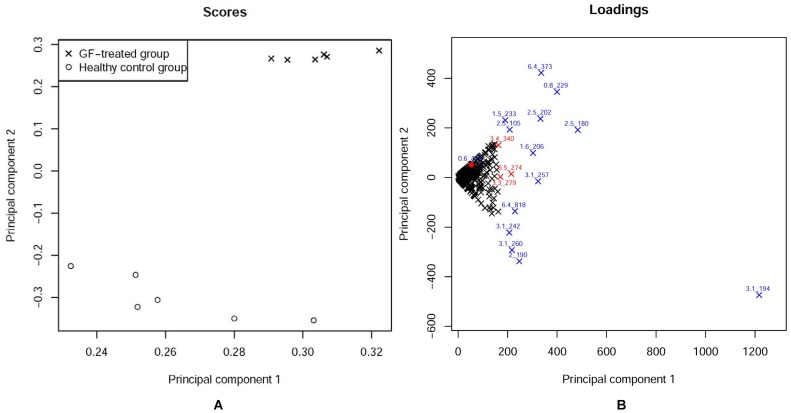
The PCA score plot and corresponding loading plot for the metabolic profiles of the GF-treated group and the healthy control group: (A) the score plot showing the separation between the GF-treated group (X) and the healthy control group (o). (B) Loading plot for potential biomarker recognition. The metabolites marked by the blue X far away enough from the origin are identified as the potential biomarkers. The metabolites marked by red X are difficult to determine whether they are the potential biomarkers. The red dot represents the retention time-m/z pair 0.6_114.

Fig. 4 presents the results of PCA of the metabolic profiles of samples of the GF-treated group and the healthy control group. It is apparent from Fig. 4A that a distinct clustering of data points from the GF-treated and healthy control groups has been achieved in the PCA score plot. Points in the PCA loading plot (Fig. 4B) stand for variables (marked by X), including their intensity and the corresponding mass-retention time pair. We selected metabolites as potential biomarkers if their corresponding points in loading plots were far away from origin, as shown in Fig. 4B.

**Figure 5:**
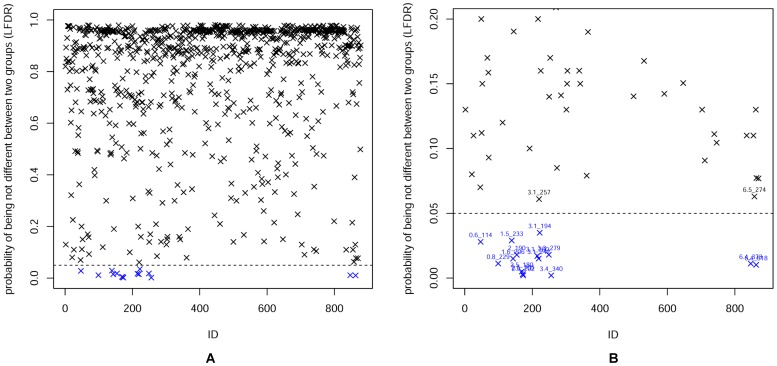
LFDR plot for the metabolic profiles of the GF-treated and healthy control groups. (A) LFDR from 0 to 1 (B) LFDR form 0 to 0.20. The metabolites marked by blue X with retention time-m/z pair below the dash line are identified as the potential biomarkers. 0.05 (dash line) is treated as the threshold for potential biomarkers identification.

We estimated the LFDR at different retention times. The LFDR plot is depicted in Fig. 5 with a threshold of 0.05 (dash line). Fig. 5A shows the LFDR from 0 to 1 for the metabolic profiles of the GF-treated and healthy control groups. In order to identify the variable marked by retention time m/z pairs clearly, the LFDR from 0 to 0.20 was presented in Fig. 5B. As seen in Fig. 5B, 15 variables presented below the dash line were selected as biomarker candidate ions with retention time m/z pairs of (3.1_194), (6.4_373), (0.8_229), (2.5_202), (2.4_180), (1.6_206), (3.1_242), (1.5_233), (2.5_105), (2.0_190), (3.1_260), (3.1_242), (6.5_818), (3.4_340) and (0.6_114). Based on the results in Fig. 5, Table 1 lists all potential biomarkers using 0.05 as a threshold. In other words, we selected a metabolite as a biomarker if its LFDR estimate was smaller than 0.05, which represents a greater than 95% probability that the m/z value of the biomarker is truly different between the two groups.

**Table 1:** LFDRs for the GF-treated group and the healthy control group at different retention times.

ID	Retention Time (min)	m/z (Da)	LFDR	1-LFDR
173	2.5	202	0.002	0.998
256	3.4	340	0.002	0.998
172	2.5	105	0.003	0.997
169	2.5	180	0.005	0.995
863	6.4	818	0.0102	0.9898
99	0.8	229	0.0112	0.9888
847	6.4	373	0.0112	0.9888
143	1.6	206	0.015	0.985
219	3.1	242	0.015	0.985
215	3.1	260	0.017	0.983
154	2	190	0.018	0.982
249	3.3	279	0.018	0.982
47	0.6	114	0.028	0.972
139	1.5	233	0.029	0.971
222	3.1	194	0.035	0.965

Potential biomarkers are shown in Table 1 and 11 of them were identified. Collision-induced dissociation experiment with collision energy varied from 10 to 30 eV was performed. We next analyzed the MS and MS/MS biomarker spectra of these candidates. We conducted searches with the MS/MS spectra in journals, the KEGG (http://www.genome.jp/kegg/), METLIN (http://metlin.scripps.edu/), SciFinder (https://scifinder.cas.org/) and HMDB (http://www.hmdb.ca/) database in order to identify the metabolites. Table 2 lists the biomarkers which correspond to the potential biomarkers shown in Table 1. Because of the lack of commercially available references and limitations of metabolite databases, not all of the potential biomarkers were identified as biomarker. The potential biomarkers (3.4_340),(3.1_260),(3.1_242) and (1.5_233) were not identified.

**Table 2:** Biomarkers of hepatotoxicity induced by GF treatment.

No	Retention Time (min)	m/z (Da)	Quasi-molecular ion	Metabolites
1	2.5	180	[M+H]^+^	Hippuric acid*
2	2.5	105		Hippurate fragment
3	2.5	202	[M+Na]^ +^	
4	2	190	[M+H]^+^	Kynurenic acid*
5	3.1	194	[M+H]^+^	Phenylacetylglycine*
		216	[M+Na]^+^	
6	1.6	206	[M+H]^+^	Xanthurenic acid**
7	0.8	229	[M+H]^+^	Leucylproline**
8	0.6	114	[M+H]^+^	citric acid*
9	3.3	279	[M+H]^+^	L-phenylalanyl-L-hydroxyproline**
10	6.4	818	[2M+H]^+^	Cholic acid*
11	6.4	373	[M+H-2H_2_O]^+^	

* Metabolite identified with database and commercially available references.

** Metabolite identified with MS/MS fragment in the database and literature.

## Discussion

Current statistical methods try to control two types of error rates: the family wise error rate (FWER) and the FDR. The Bonferroni correction method is a well-known p-value correction method that is widely used to control the FWER during multiple hypothesis testing. This correction method is simple and easy to carry out. However, the Bonferroni correction method is too conservative. It is also difficult to detect biomarkers if there are many candidate metabolites (e.g., more than ten metabolites) taken into consideration. The FDR is the expected ratio of the number of false positives over the total number of rejections of the null hypothesis. In 1995, Benjamini and Hochberg [24] introduced a procedure to determine the null hypothesis rejection of each hypothesis comparison. The BH method provides more statistical power than the Bonferroni method. Nevertheless, the FDR as a measure cannot be assigned to each hypothesis comparison. For instance, we cannot estimate the FDR value for each candidate metabolite when attempting to detect metabolic biomarkers. Fortunately, the LFDR can solve this problem and be assigned to each candidate metabolite.

Based on Fig. 2B, metabolites 4, 6, 9, 11 and 12 were easily identified as potential biomarkers. In contrast, the other biomarkers (i.e., the number in red except 4, 6, 9, 11 and 12) were difficult to be distinguished from other markers (i.e., the numbers in black) because both biomarkers except 4, 6, 9,11 and 12 and other markers were mixed together. Nevertheless, using the LFDR method, we were able to detect 11 in 15 biomarkers correctly by setting 0.05 as a threshold (see Fig. 3). Moreover, we were able to identify all the biomarkers by increasing the threshold to 0.1. But some biomarker candidates which were not designed as biomarkers were detected. However, the percentage of incorrect detection by using LFDR is lower than that by using PCA.

Considering the metabolic profiles of the GF-treated and healthy group data, as shown in Fig. 4B, 13 variables far away from the center were selected as biomarker candidate ions (see the results section). In contrast, for the 3 variables marked by (6.5_274), (3.3_279) and (3.4_340), it was difficult to determine whether they were far enough away from the origin or not. However, when we set the threshold of the LFDR method to 0.05, meaning that the probability that the m/z value of a biomarker was truly different between the two groups must be at least 95%, then 15 variables were detected as potential biomarkers including 3.4_340 (shown in Fig. 5 and Table 1). The retention time-m/z pair of 0.6_114, a biomarker “citric acid” shown in Table 2, was detected by using the LFDR method, but not the PCA method (Fig. 4B). Thus, potential biomarkers can be detected more precisely by using the LFDR method than the PCA method.

### Conclusions

Based on PCA method, researchers only focus on points in the loading plot that represent metabolites and determine that a metabolite is a potential biomarker if its corresponding point in the loading plot is far away enough from the origin. However, the decision of what distance should be used as a cutoff is based on the discretion of the researcher. Therefore, PCA cannot provide quantitative evidence to support potential biomarker detection. The LFDR in biomarker detection problems presents the posterior probability that the m/z value of a biomarker is truly different between experimental and control groups. It provides a quantitive method to evaluate how likely a biomarker candidate is a biomarker. Furthermore, based on biomarker detection in the simulation data and the metabolic profiles of the GF-treated and healthy group data, we were able to use the LFDR method to detect some potential biomarkers that were difficult to be identified by the PCA method. Thus our data suggests that the use of LFDR for metabolite biomarker identification is practical and accurate.
